# Identifying patient preferences for diabetes care: A protocol for implementing a discrete choice experiment in Samoa

**DOI:** 10.1371/journal.pone.0295845

**Published:** 2023-12-22

**Authors:** Anna C. Rivara, Omar Galárraga, Melania Selu, Maria Arorae, Ruiyan Wang, Kima Faasalele-Savusa, Rochelle Rosen, Nicola L. Hawley, Satupaitea Viali

**Affiliations:** 1 Department of Epidemiology (Chronic Diseases), Yale School of Public Health, New Haven, Connecticut, United States of America; 2 Department of Health Services Policy and Practice, and International Health Institute, School of Public Health, Brown University, Providence, Rhode Island, United States of America; 3 Obesity Lifestyle and Genetic Adaptations (OLaGA) Research Center, Apia, Samoa; 4 Centers for Behavioral and Preventative Medicine, The Miriam Hospital, Providence, Rhode Island, United States of America; 5 Department of Behavioral and Social Sciences, Brown University, School of Public Health, Providence, Rhode Island, United States of America; 6 School of Medicine, National University of Samoa, Apia, Samoa; Public Library of Science, UNITED KINGDOM

## Abstract

In Samoa, adult Type 2 diabetes prevalence has increased within the past 30 years. Patient preferences for care are factors known to influence treatment adherence and are associated with reduced disease progression and severity. However, patient preferences for diabetes care, generally, are understudied, and other patient-centered factors such as willingness-to-pay (WTP) for diabetes treatment have never been explored in this setting. Discrete Choice Experiments (DCE) are useful tools to elicit preferences and WTP for healthcare. DCEs present patients with hypothetical scenarios composed of a series of multi-alternative choice profiles made up of attributes and levels. Patients choose a profile based on which attributes and levels may be preferable for them, thereby quantifying and identifying locally relevant patient-centered preferences. This paper presents the protocol for the design, piloting, and implementation of a DCE identifying patient preferences for diabetes care, in Samoa. Using an exploratory sequential mixed methods design, formative data from a literature review and semi-structured interviews with n = 20 Samoan adults living with Type 2 diabetes was used to design a Best-Best DCE instrument. Experimental design procedures were used to reduce the number of choice-sets and balance the instrument. Following pilot testing, the DCE is being administered to n = 450 Samoan adults living with diabetes, along with associated questionnaires, and anthropometrics. Subsequently, we will also be assessing longitudinally how preferences for care change over time. Data will be analyzed using progressive mixed Rank Order Logit models. The results will identify which diabetes care attributes are important to patients (p < 0.05), examine associations between participant characteristics and preference, illuminate the trade-offs participants are willing to make, and the probability of uptake, and WTP for specific attributes and levels. The results from this study will provide integral data useful for designing and adapting efficacious diabetes intervention and treatment approaches in this setting.

## Introduction

Type 2 diabetes mellitus (hereafter referred to as diabetes) is expected to affect 592 million people by 2035 [1]. Low- and middle-income countries (LMICs) are disproportionately impacted–the Pacific Islands more so than any other region [2]. In Samoa, adult diabetes prevalence increased between 1978 and 2013 from 1.2% to 19.6% in men, and 2.2% to 19.5% in women [3]. In a cohort of adult Samoans (n = 500) diabetes prevalence more than doubled from 11.1% to 27.8% in men and 13.1% to 29.0% in women between 2010 and 2018 [4]. For comparison, 9.3% of adults (≥ 20 years) globally were estimated to have diabetes in 2019 [5]. In 2009, close to 20% of households in Samoa had at least one person who had been diagnosed with diabetes [6, 7].

Work in other settings shows that patient preferences for care are known factors influencing treatment and indirectly (through sustained engagement in care) are associated with reduced disease progression and severity [8, 9]. Health interventions that are culturally adapted, context-specific, and/or incorporate user preferences have been shown to have high impact [10–16] and are better able to reach and improve health outcomes in populations that are at high risk and underserved [17, 18]. Patient preferences, generally, for diabetes care are understudied, and the main factors that guide patients’ decisions to maintain treatment adherence remain largely unidentified. Additionally, other patient-centered factors, which are important for increasing the efficacy and acceptability of care [8, 9, 19, 20], are not well examined in this setting. For example, willingness-to-pay (WTP) [21, 22], which reflects what patients may expect and value from their diabetes care, has yet to be explored in Samoa.

Patient preferences and WTP for improvements in diabetes treatment have been assessed using Discrete Choice Experiments (DCEs) in the US [23], the Netherlands [24] and Denmark [9], but few published studies have used DCEs to measure diabetes care preferences in low- and middle-income countries (LMICs) [25]. These specialized surveys present participants with hypothetical scenarios composed of a series of multi-alternative choice profiles. The alternative profiles are made up of attributes (healthcare components/characteristics such as cost, treatment side effects) with different continuous or categorical levels (e.g., differing treatment prices, binary presence, or absence of side effects, etc.). Participants are asked to choose a profile based on what attribute levels may be preferable for them. Therefore, DCEs add to qualitative measures by capturing quantitative data on strength of preferences and the probability of uptake of defined choices [26]. As a survey- and attribute-based measure of value that provides stated preference data (measuring what participants say they *will do* versus what they *do/have done*), DCEs are particularly useful in settings where patient care experiences are understudied [26].

This project will identify diabetes care attributes that are important and valued by Samoan adults living with diabetes. Additionally, results from this project will provide valuable data to the health system regarding patients’ probability of uptake and WTP for potential services. Finally, the results from this project will provide the formative data necessary to design and adapt efficacious and patient-centered diabetes intervention and treatment approaches.

## Materials and methods

### Aims and hypotheses

**Fig 1:**
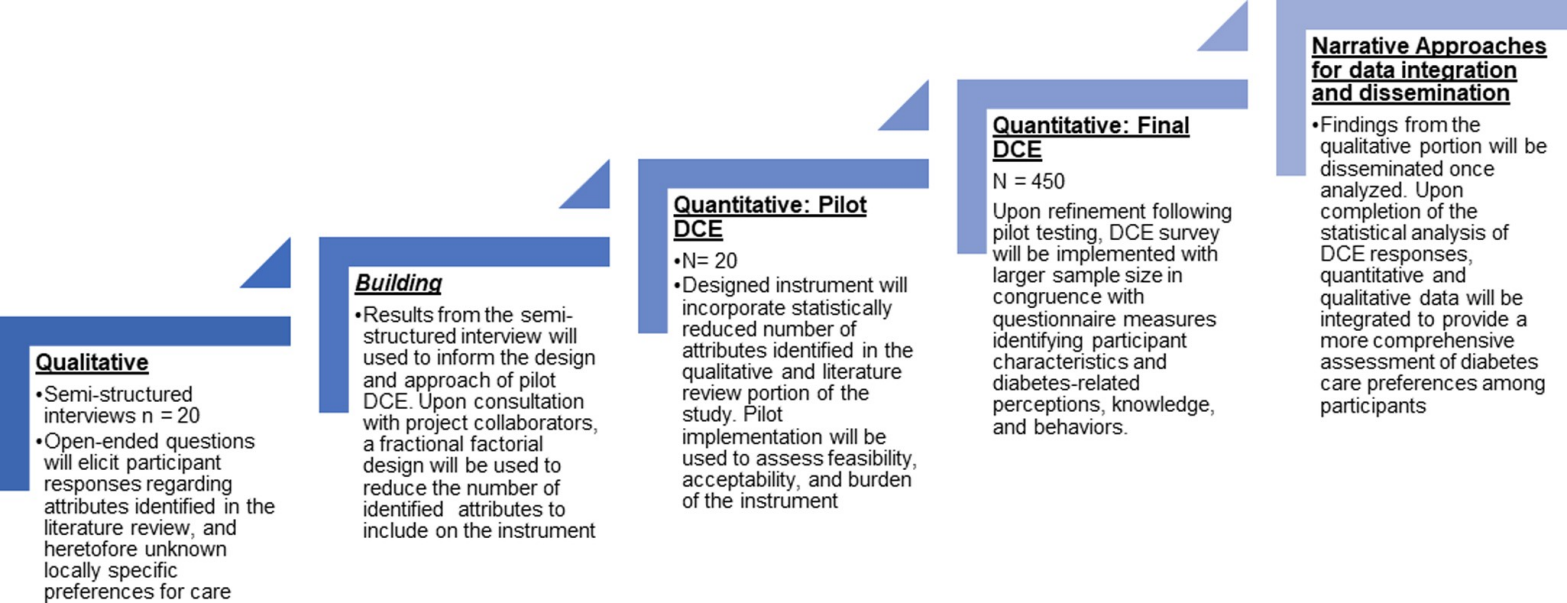
Exploratory sequential design.

Using an exploratory sequential mixed methods approach (Fig 1) [27, 28], we will design, develop, pilot, and administer a DCE in Samoa to identify preferences for diabetes care.

The aims of this project are:

1. To design, pilot, and implement a DCE measuring diabetes care preferences in Samoa.
2. To identify and measure the strength of preference and importance of attributes of diabetes care alternatives and standard care options.
3. To identify the trade-offs (i.e., marginal Willingness to Pay (WTP)), participants are willing to make to obtain preferred attributes, and the probability of uptake for such services.
4. To measure longitudinal changes in care preferences over time.

### Setting

Samoa is an independent, lower-middle income country in the Western Pacific. The majority (>80%) of the estimated population of 206,179 [29] reside on the island of ‘Upolu. ‘Upolu is made up of three census regions: the Apia Urban Area (AUA; Apia is the capital of Samoa), Northwest ‘Upolu (NWU; peri-urban) and the Rest of ‘Upolu (ROU; rural). A smaller proportion of the population lives on the more rural island of Savai’i (SAV census region). Recruitment will be taking place on both islands. The Samoan healthcare system has been characterized as hospital-centric [30] and largely centralized in Apia, where one of two referral hospitals in the country is located (the other on Savai’i). Throughout ’Upolu and Savai’i, there are approximately 12 Ministry of Health-run regional hospitals, and clinics [30]. However, there is only one designated diabetes clinic, centrally located in Apia, where many diabetes patients nationally are referred for treatment. During care encounters, patients generally receive assessments of weight, height, abdominal circumference, blood pressure and capillary blood glucose. Doctors provide counseling regarding medication adherence, diet and physical activity, hypoglycemia symptoms, as well as provide referrals to the hospital eye clinic for detailed fundoscopy, if needed (Viali, personal communication). However, the level of detailed diabetes-related care, and diabetes-related education provided for patients can be variable based on resource limitations. Decentralization of the health system and an increasing focus on primary care utilization, health promotion and prevention, community empowerment, and a specific focus on noncommunicable disease management are currently being undertaken as part of the Samoan Government’s NCD Policy and Action Plan (2019–2029) and an ongoing World Bank-led Health Systems Strengthening Program [30].

### Sample size and inclusion criteria

**Fig 2:**
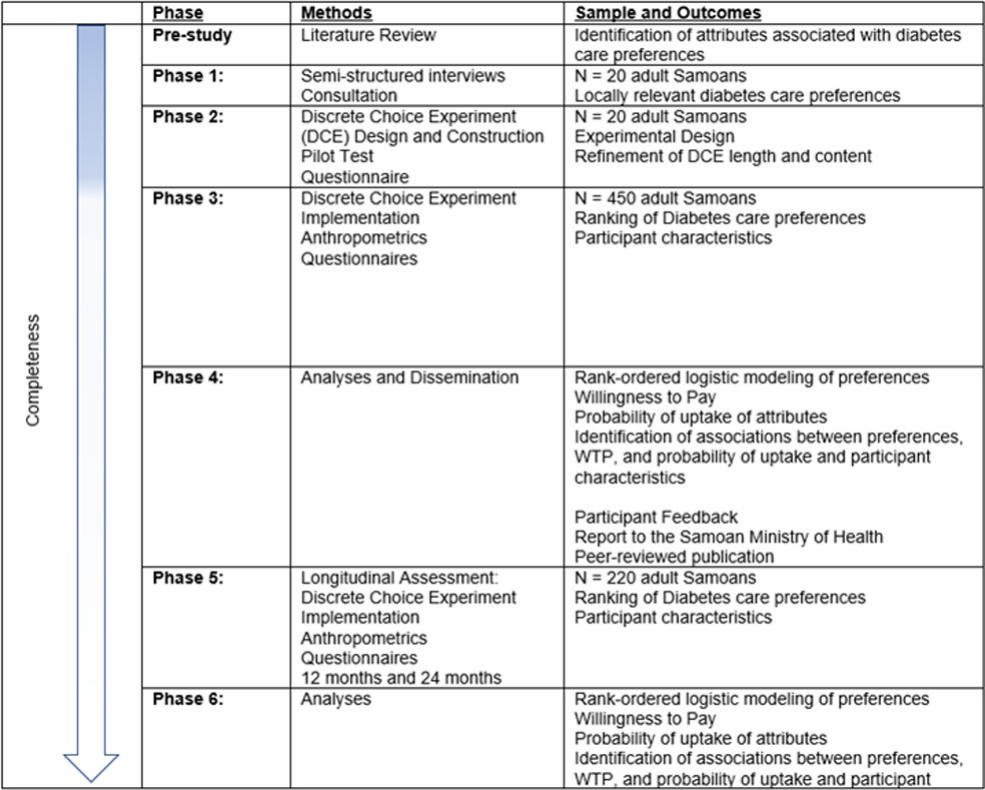
Timeline.

Participants in all phases of the study will be adult Samoans (30 to 80 years) recently diagnosed with diabetes (<12 months for the pilot, <24 months for the finalized implementation) who provide permission to access their medical records and are willing to provide written informed consent for study participation. Twenty participants completed semi-structured interviews and were administered the pilot DCE (completed July 2023). Following refinement of the instrument, the finalized DCE survey is now being administered to n = 450 Samoan adults living with diabetes, along with associated questionnaires and anthropometric measures (initiated August 2023). Baseline recruitment is expected to be completed by July 2024. Two hundred and twenty participants will complete additional DCEs at 12- and 24-months post baseline assessment (Fig 2). Potential participants will be excluded if they are unable or unwilling to complete study protocols and/or were only diagnosed as having gestational diabetes (given the unique healthcare needs of pregnancy). Equal numbers of men and women (50.0%) will be recruited for each phase of the study. Participants will be recruited from regional Ministry of Health run regional hospitals and clinics throughout the islands of ’Upolu and Savai’i, and the diabetes specialty care clinic located centrally in Apia. Participants will also be recruited using the Obesity Lifestyle and Genetic Adaptations (OLaGA) Research Center’s social media page (>9,700 followers). The OLaGA Research Center is a collaboration between Yale and Brown Universities and the Samoan Ministry of Health. After determining eligibility, Samoan Research Assistants (RAs) will introduce the study objectives to the participants. Before each study assessment, written informed consent will be obtained from each participant.

### DCE instrument construction

The design and construction of the DCE survey was based on formative data collected from a literature review and semi-structured interviews following best practice recommendations [26, 31, 32]. We also followed the components of the 10-item checklist developed by the International Society for Pharmacoeconomics and Outcomes Research (ISPOR) Good Research Practices for Conjoint Analysis Task Force to guide the design, development, and implementation of the instrument [33]. Briefly, the 10-items include: 1) the formation of the research questions, 2) identification of attributes and levels, 3) construction of choice sets, 4) experimental design of the instrument (statistical procedures to reduce the number of attribute and level combinations and increase the precision of the survey) 5) preference elicitation, 6) instrument design, 7) data collection plan, 8) statistical analyses, 9) results and conclusions, and 10) study presentation [33]. This project is guided by two main research questions: 1) What attributes of diabetes care do adult Samoans living with diabetes value and prefer, and how do these compare to attributes currently available within standard care protocols? and 2) what are the trade-offs Samoan adults living with diabetes are willing to make to obtain these attributes or services?

### Literature review

We reviewed the existing literature to identify potential attributes for inclusion on the DCE. Papers were included if they focused on adults (age restricted to individuals 18 years and older), were in English, and described care for diabetes (including both Type 1 and Type 2 diabetes, as well as prediabetes management). Databases including PubMed, Medline, and Google Scholar were searched with terms comprised of: “diabetes care attributes”, “diabetes care preferences”, “diabetes care barriers”, “diabetes care adherence”, and “‘Diabetes’ and ‘DCE/Discrete Choice Experiment’”. Study type was not restricted; therefore, results were obtained from studies that used DCEs, quantitative and qualitative interviews, cross-sectional surveys, systematic reviews, and randomized control trials. The review included 45 papers, 22.2% of which focused specifically on DCEs. Attributes identified as important for and/or influencing diabetes care adherence included: intervention/treatment setting [34]; type of provider (i.e., doctors, nurses, educators, case managers, community health workers) [34–37]; provider/healthcare interactions (i.e., service dissatisfaction [38], as well as providers’ cultural competency, consistency of communication, transparency, respect, and compassion) [37–45]; shared decision-making [36, 44]; levels of diabetes education and knowledge provided [39, 41, 45–48]; treatments that improve quality of life [44, 49, 50]; medication/treatment side effects [23, 50–56]; treatment outcomes/goals [9, 23, 49, 52, 53, 56–58]; un- and under-coordinated care [39, 59]; types of self-management and regimen frequency/complexity [34, 36, 40, 43, 48, 50, 53, 54, 60]; access to care and resources [40, 41, 45, 61]; family and social support [42, 59, 62]; emotional support [47]; time requirements [45, 47, 61–64]; and cost/financial support [41, 55, 56, 59, 61].

### Semi-structured interviews

Semi-structured interviews were conducted with n = 20 adults (30–80 years) living with diabetes in Samoa (diagnosis < 12 months). Semi-structured interviews, the construction of the DCE, and piloting of the instrument were funded by the Yale Institute for Global Health’s Global Health Spark Award. Analysis of these interviews resulted in the refinement of the list of attributes and levels used to design the pilot DCE survey [27, 28]. Interviews explored participants’ diabetes care experiences (to contextualize their responses), their preferred attributes and levels of diabetes care (capturing what is important to them and what facilitates their retention in care), and their current care experiences (to inform the standard care option). Interviews were conducted by Samoan RAs trained in qualitative methods (in either Samoan or English based on participant preference) with the goals of (1) refining the list obtained from the literature review and (2) identifying other unlisted attributes that have local relevance. This exercise was used primarily to capture preferences that lie *outside* the domains recognized clinically or generally by the literature review [65]. Additionally, the interviews informed the assignment of appropriate levels to the attributes (e.g., range of acceptable costs for care, treatment locations, etc.). Following transcription and translation of recorded interviews by the RAs, interviews were double coded by members of the study team; NVivo [66] was used to support data analysis. Content analysis was used to identify initial themes regarding diabetes care attribute and level preferences. Additional dating mining tools–such as word and compound query frequencies–were used to identify the most frequently shared and important attributes. Subsequently, consultation with project investigators (Viali) and feedback from the Samoan Ministry of Health’s Health Research Committee regarding feasibility of implementation for identified attributes, facilitated the design of the pilot DCE instrument [32, 67–71].

### Choice set design, and preference elicitation

A ‘Best-Best’ DCE was constructed (*preference elicitation)*. Best-Best DCEs elicit additional preference information, are designed to inherently rank choices, and increase the statistical power for analyses [72–74]. Participants are being presented with a series of choice sets, each with three different alternative care profiles (one of which will always be an ’opt-out/none of these’ for each attribute). Each profile presents a hypothetical scenario (comprised of combinations of attributes and levels) for diabetes care. Participants are asked to choose their most preferred option from the three profiles, followed by their second preference (but not worst) from the remaining alternatives. Because treatment protocols vary by patient, it was challenging to identify a true ’standard care’ level for each attribute. Frequently cited experiences (e.g., only being able to see the first available doctor) were included as potential levels in the design of the instrument and are intended to represent attribute levels generally (but not inclusive to all) experienced by patients. Our aim in doing so is to facilitate a comparison of the preferences for alternative options compared to these commonly experienced encounters; additionally, the inclusion of an opt-out option, provides better representation of real-world scenarios [72].

To design the alternative profiles for both the pilot and final instrument, we mirrored similar DCEs utilized elsewhere and included 8 of the most frequently reported attributes (garnered from the literature review, interviews, and consultation) in the instrument (DCEs within health economics are estimated to have a mode of 6 attributes but can range from 2–24 [31]). The eight attributes included in the pilot was reduced to seven following refinements. Each attribute is between 3–6 levels. The chosen attributes and levels were entered into JMP^®^ Pro 15 (SAS Institute Inc., Cary, NC) where we specified the creation of 12 choice sets for the pilot, and 8 choice sets for the final baseline DCE [71]. Limiting the number of attributes, levels, and choice sets was undertaken to prevent participant fatigue and reduce cognitive burden [32, 73]. We utilized a fractional factorial design [26, 31, 32] to reduce the high number of possible combinations of attributes and levels that could be included in each choice set and an efficiency design to maximize precision [23, 75] (*experimental design*). Both the pilot and final DCE included continuous (i.e., costs of treatment) and categorical attribute levels. Attribute levels are balanced and appear an equal number of times (reducing bias). Further, JMP software allows for the randomization of the order of the choice sets that are presented to the participants and for the creation of multiple survey blocks (we created 9 survey blocks). This helps to ensure the random elicitation of choice sets (reducing bias) and increases the diversity of attribute and level combinations that are encountered by the participants. Block assignments will be adjusted for during analysis.

### Pilot testing

The DCE survey was pilot-tested and refined before initiating implementation among the full sample of participants. The goal of piloting the instrument was to determine participant understanding of the attributes, levels, and choice-sets, as well as their comfortability with the length of the DCE. The DCE was translated into Samoan and administered by the RAs to participants using Qualtrics software [76] for tablet-based data collection. The same n = 20 adults who participated in the semi-structured interviews were asked to complete the pilot survey. Participants evaluated the survey with regards to length, satisfaction, relevance, and comprehension. After finalizing and refining the pilot survey, we designed the finalized DCE instrument (9 survey blocks, 8 choice sets per survey). The length of each survey, and the number created through the fractional factorial design precluded the inclusion of each in this manuscript. Instead, the included attributes and levels used to design the finalized instrument can be found in the supporting information section (S1 Table).

### Implementation

The finalized DCE instrument will be administered to n = 450 adult Samoans recently diagnosed with diabetes (data collection started August 2023; funded as an aim of 5K01DK132514: The Diabetes Clinical outcomes Associated with Retention and Engagement in Care, in Samoa (Diabetes CARE); PI: Rivara). After obtaining written informed consent from all participants, RAs are explaining the study objectives, describing what a DCE is (i.e., an explanation that the presented choice sets represent hypothetical scenarios), and are consistently describing the intended definitions of the included attributes and levels. This latter step is intended to help participants assess levels that may be subjective or unfamiliar. For example, continuous glucose monitoring is an included attribute level. This type of glucose management is not common in Samoa at this time, and further explanation may be needed for some participants [33]. In addition to administering the DCE, RAs are collecting demographic (i.e., sex, age, region of residence), anthropometric (i.e., weight and height, and blood pressure), health, and socioeconomic data (income and assets) from participants. Participants are also being administered diabetes knowledge [77], self-management and distress, and treatment adherence [78] questionnaires. These data will be used to examine whether attribute preferences vary by sex and/or are associated with chronic disease risk, age, socioeconomic position, or levels of diabetes knowledge. Assessments take approximately 30–40 minutes.

Two hundred and twenty participants will be asked to complete additional DCEs at 12- and 24-months post initial assessment. The same DCE instrument will be used to measure how preferences change over time as individuals live and manage their diabetes.

### Planned statistical analyses

The statistical modeling of the DCE will be based on random utility theory, where participants choose between alternative profiles, and the attribute levels of the favored option is associated with the highest utility (benefit) to the specific participant [79]. A mixed logit model will be used to estimate choice models from the ’first best’ data and to observe the direction of the rank-ordered attribute choices [74, 80]. Rank order logit (ROL) modeling or Exploded Logit Modeling [81–83] will be used to observe a full ranking of the alternatives in all choice-sets (including first-best and second-best options in the same model) [74] and determine the probability that a participant chooses one option over the others, based on the chosen option’s attribute levels, while controlling for random factors and interindividual tastes [26, 71]. These types of analyses are useful for identifying unknown/unobserved heterogeneity of preferences [26, 73, 84] and will allow for analysis of the cross-sectional panel-like data (participants will be completing multiple choice sets and there will be multiple responses per case/participant). Progressive mixed ROL models will be constructed [74]. A mixed logit version of the ROL will be estimated by ’exploding’ the data as outlined in Lancsar et al. (2017) [72]. Briefly, this procedure will allow each choice set to result in five rows of data, with the first three rows (first sub-choice set) indicating the first-best preference (coded as 1, the remaining two alternatives as 0) and the remaining two rows (second sub-choice set) indicating the second-best preference (coded as 1, the remaining alternative as 0) [72]. By organizing the data in this manner, a traditional mixed multinomial logit model can be conducted that assesses the ranked choices [72]. The chosen profiles will be the dependent variable, and the levels of the attributes will be the independent variables (standard care attribute levels will be initially coded as the reference variable). Interaction terms will be introduced in Model 2 to estimate any associations between attributes and sex. Other case-specific characteristics (e.g., age, levels of diabetes knowledge, weight status, etc.) will be interacted with the intercept and selected attributes to explore the effect of participant characteristics on preferences in Model 3. [74]. We plan to use dummy coding (rather than effects level coding) for categorical variables to facilitate ease of interpretation [73, 74, 85]. The models will allow for the identification of which attributes are important (βs with p<0.05; Aim 2), the direction of that importance (positive or negative βs), and the relative importance of attribute levels compared to others (ratio of estimated parameter to relevant comparator; Aim 2). Further, we will be able to illuminate the trade-offs participants are willing to make between attributes, including marginal WTP (Aim 3). Because we will be including cost of treatment as an attribute, we can estimate the marginal WTP for other attributes; generally, marginal WTP can be estimated by dividing the estimated utility coefficient of an attribute by the utility coefficient of the cost of treatment attribute, holding other attributes constant [9, 86]. We will also be able to estimate the probability of participants taking up a care profile with specific attributes [26]. Sensitivity analyses will be undertaken if we suspect that the relative marginal utility of attributes varies due to differences in income levels. This may include exploring whether preference weights, probability of uptake of alternatives, and WTP vary by different income levels (Satisfaction from the attribute may be based on its associated costs; e.g., perceived satisfaction/preferences of care options may differ by participants’ ability to pay compared to their peers) [84, 87]. The distribution (level balance) of the attributes will be checked across the first and second preferred profiles to ensure the DCE is balanced and that logit model properties are met [88, 89].

Few longitudinal studies using discrete choice experiments have been undertaken [90], therefore analyses will be largely exploratory. Because time between assessments (12 months) is short, the same DCE instrument will be used at each timepoint [90, 91]. Following best practices, we will analyze models at each timepoint using multinomial logit modeling [91]. The Chow Test Analogue for logistic regression will be used to test whether the models’ attribute coefficients significantly differ between timepoints. The Chow Test Analogue allows for comparative analysis of different regression models fitted to two different data sets [91]. By including demographic, socioeconomic, and care-related questionnaire data into our models, we will be able to explore how external factors, and especially changes in external factors such as income status, care use/disuse, and diabetes management and distress, are associated with changes in preferences over time. In the unlikely case that we experience greater loss to follow-up and attrition than expected, we will use repeated cross-sectional DCE approaches (such as a covariate extended model) to measure if differences in preferences across samples are significant [90]. Analyses will be completed in Stata v. 17 (College Station, TX) using the Choice Models (CM) suite of commands [92].

### Sample size and power

The minimum sample size (*N)* required for DCEs can be calculated as *N≥ (500 x c)/(TxA)*, where T is the number of included choice sets, A is the number of alternative profiles per set, and c is the number of analysis cells (attributes). C can be equal to the greatest number of levels for any attribute to assess main effects [31, 73]. The final DCE includes 8 choice sets (T), 7 attributes, 6 levels (C) and 3 alternatives (including the opt out option; A); the minimum number of participants needed is n = 125. To explore heterogeneity by factors such as sex, age, or residence, to increase the statistical power of our analyses, and to mirror other DCE studies [31], our aim is to recruit a larger sample size.

### Dissemination of results

Results of the study will be presented to participants and the Samoan Ministry of Health upon completion of the study. Additionally, we will disseminate the findings to peer-reviewed journals. We plan to use a staged narrative approach to disseminate the findings. Interview data will be published upon analysis [28]. Integration of the qualitative and qualitative results will likely occur in the quantitative-focused papers following the analysis of the DCE (using a narrative weaving approach to report on the findings of the interviews and DCE while also mapping how the DCE attributes corresponded to the interview responses [28]). We plan to also incorporate joint displays [28] to integrate the qualitative and quantitative data to present the results at academic conferences and invited seminars.

### Project status and ethical considerations

This project has received ethical approval from the Yale Institutional Review Board (Yale IRB 2000031387; 2000032062) and the Health Research Committee at the Samoan Ministry of Health. Enrollment for the finalized baseline DCE assessment was initiated August 2023 (Fig 2).

### Inclusivity in global research

Additional information regarding the ethical, cultural, and scientific considerations specific to inclusivity in global research is included in the supporting information section (S1 Checklist).

## Discussion

Effective engagement in care is critical to reduce the physiological, economic, and social burdens of diabetes [2, 3, 93]. In Samoa, however, patient preferences for diabetes care or how care can be best optimized to facilitate patient retention is understudied. The present protocol aims to address this knowledge and practice gap and inform future diabetes care approaches. By conducting a DCE, we will be able to identify and quantify preferences Samoan adults living with diabetes have regarding their diabetes care and provide policy-relevant estimates of their willingness to pay for and engage in these services.

The timing of this study and its expected outputs are aligned with the implementation of a World Bank-funded, five year, NCD-focused health system strengthening project [94] and the development of the next Samoa National NCD policy [95], in which the government plans to invest heavily in preventative care strategies and secondary prevention. This project will provide vital information regarding the design of diabetes care programming options that may increase the likelihood of care engagement and retention. Samoan leadership in regional NCD networks means that the methodological approach and results of the work can be shared to shape regional practice and policy throughout the Pacific and beyond. Given that Samoan cultural traditions and behavioral norms are shared among Samoans throughout the diaspora (and many Pacific Islanders more broadly) we expect that our results may also be applicable and integral to reducing the high and disproportionate burden of diabetes experienced among Samoan and Pacific Islander populations [96–99]. Our work aims to identify critical, evidence-based, and culturally acceptable care solutions that will engage and retain diabetes patients in treatment and provide them with greater agency in their disease management.

We recognize several potential limitations to the presented protocol. As a survey-based method, DCEs are limited in their ability to capture the individualized lived experiences of having diabetes and/or the more comprehensive litany of preferences that people living with diabetes have regarding their care. Nonetheless, we believe that the potential this method holds for providing novel and representative information about diabetes care preferences in the country transcends its limitations. Additionally, by using semi-structured interviews to design the instrument, our aim was to capture potential Samoan-specific preferences for care that will facilitate the ultimate construction and adaptation of locally relevant treatment options. We are also restricting our sample to individuals recently diagnosed with diabetes to control for changes in care preferences related to disease duration and progression [36, 100]. While our longitudinal analyses aim to capture how care preferences may change over time, a longer time examination of those preferences is needed.

The approach described here is designed to generate impactful, policy and practice-relevant data using a discrete choice experiment. The results obtained will provide important documentation of diabetes care preferences among Samoans. These findings have the potential to shape diabetes treatment design, adaptation, and implementation within high-need settings and among high-risk populations.

## Supporting information

S1 Table Attributes and levels included in discrete choice experiment survey design. (DOCX)

S1 Checklist Inclusivity in global research checklist. (DOCX)
