# Supplementary material for: Identifying patient preferences for diabetes care: A protocol for implementing a discrete choice experiment in Samoa
Source: PLoS One. 2023 Dec 22;18(12):e0295845. doi: 10.1371/journal.pone.0295845 (PMC10745180; doi:10.1371/journal.pone.0295845)
Supplement: S1 Table — (DOCX) [file pone.0295845.s001.docx]

Supplementary Table 1: Attributes and Levels used to design the DCE Survey

| **Attribute** | **Levels^[[1]](#footnote-1),^^[[2]](#footnote-2)^** |
| --- | --- |
| Location | National hospital |
|  | District hospital |
|  | Private Clinic |
|  | Specialty Diabetes Clinic |
|  | Regularly scheduled village-based mobile clinics |
| Medication availability | National Hospital Pharmacy |
|  | District Hospital Pharmacy |
|  | Privately Owned Pharmacy |
| Appointment Day and Time | Weekday Mornings 8am – 1pm |
|  | Weekday Afternoons 2pm - 4 pm |
|  | Weekday Evenings 5pm – 8pm |
|  | Saturday Mornings 8am – 1pm |
|  | Saturday Afternoons 2pm – 4pm |
|  | Saturday Evenings 5pm – 8pm |
| Treatment Management and Support | Standard information about diets, medications, and symptoms |
|  | Additional information provided by a Traditional medical practitioner |
|  | Additional information about coping, mental health, and dealing with emotions delivered by a mental health specialist |
|  | Additional information about diets delivered by a dietitian |
| Preferred Provider | First available doctor |
|  | First available nurse. |
|  | Mobile doctor or nurse (comes to villages and homes) |
|  | Nurse who only sees diabetes patients |
|  | Doctor who only sees diabetes patients. |
| Innovation | Continuous glucose monitoring |
|  | Usual treatment appointments with providers to discuss diabetes progression, and questions |
|  | Diabetes support within a group environment |
|  | Text reminders for self-management (appointment and refill reminders) |
|  | Chat/text message help lines |
|  | Telehealth (talk with doctors over videoconferencing calls) |
| Cost | You pay $40 WST |
|  | You pay $20 WST |
|  | You receive $40 WST |
|  | You receive $20 WST |

1. Participants are shown three alternative profiles within each choice set. Attribute levels for profiles A and B are each comprised of one the above options. Additionally, participants are presented with Choice Profile C/none of these, which consisted of an opt-out/none of these option for each attribute (with $0 WST included as the Choice C cost level). Participants are then asked to choose their most preferred choice profile [A, B, or C (none of these)], and then their second most preferred from the remaining profiles. [↑](#footnote-ref-1)
2. The attributes and levels within each of the choice sets presented to participants is provided in Samoan and English. [↑](#footnote-ref-2)
