# Supplementary material for: Identifying patient preferences for diabetes care: A protocol for implementing a discrete choice experiment in Samoa
Source: PLoS One. 2023 Dec 22;18(12):e0295845. doi: 10.1371/journal.pone.0295845 (PMC10745180; doi:10.1371/journal.pone.0295845)
Supplement: S1 Checklist — (DOCX) [file pone.0295845.s002.docx]

Inclusivity in global research

PLOS’ policy on inclusivity in global research aims to improve transparency in the reporting of research performed outside of researchers’ own country or community and ensures that PLOS publications reporting global research adhere to high standards for research ethics and authorship. Authors of relevant research articles may be asked to complete the questionnaire below, which outlines ethical, cultural, and scientific considerations specific to inclusivity in global research. This questionnaire may be requested when researchers have travelled to a different country to conduct research, if research uses samples collected in another country, research with Indigenous populations or their lands, or if research is on cultural artefacts. Researchers travelling to another country solely to use laboratory equipment will not normally be required to complete the questionnaire. However, the questionnaire can be requested at the journal’s discretion for any submission – if you have been requested to complete this questionnaire by the PLOS journal you submitted to, please do so.

Please complete the questionnaire below and include this as a Supporting Information file with your manuscript. Note that if your paper is accepted for publication, this checklist will be published with your article in the supporting information files. Please ensure that you reference the checklist in the main body of your manuscript. We suggest adding a subsection ‘Inclusivity in global research’ to your Methods section and adding the following sentence: “Additional information regarding the ethical, cultural, and scientific considerations specific to inclusivity in global research is included in the Supporting Information (SX Checklist)”

The questions have been designed to be applicable to a wide range of study types, and there are subsections for both human subjects research and non-human subjects research. If any of the questions are not relevant to your research please mark them as “N/A” as appropriate.

**Ethical considerations, permits and authorship**

*This section is applicable to all research types.*

Provide details as to who granted permissions and/or consent for the study to take place in the Methods section of your manuscript. This should include the names of **all** ethics boards, governmental organizations, community leaders or other bodies that provided approval for the study. If individuals provided approval refer to these people by their role or title but do not list their name(s).

Reported on page number: 13

If there were any deviations from the study protocol after approval was obtained please provide details of these changes in the Methods section of your manuscript.
Did this study involve local collaborators that are residents of the country where the research was conducted or members of the community studied? If you do not have any authors from said communities, please provide an explanation for this below.

Reported on page number: N/A

Yes. Co-authors MS, MA, and KFS are Samoan researchers and part of the Yale OLaGA research group. They were responsible for leading pilot data collection, and will be leading the data collection for the final project. They are all actively involved in the project design, and will be contributing to the analyses of the data. Senior author SV is the first author's K01 mentor and a consultant, supervisor, and collaborator on the project design and project implementation.

Everyone listed as an author should meet PLOS’ criteria for authorship and all individuals who meet these criteria should be included in the author byline, rather than the acknowledgements. For further information please see the journal’s Authorship Policy.

**Human subjects research (e.g. health research, medical research, cross-cultural psychology)**

Did you obtain written informed consent from a representative of the local community or region before the research took place? How did you establish who speaks for the community? Details of written informed consent obtained from study participants should be reported separately in the Methods section of your manuscript.

Yes. Ethical research permission was gained from the Samoan Ministry of Health's Health Research Committee (the IRB of record for all health research conducted in Samoa). All participants who were engaged in the pilot phase of the study provided written informed consent, and participants for the final implementation will provide written informed consent. This information is also provided in the manuscript.

How did members of the local community provide input on the aims of the research investigation, its methodology, and its anticipated outcome(s)?

Co-authors MS, MA, KFS, and SV have all actively collaborated with our team from between 5 – 20 years. Our research is guided by their input regarding the needs of the local community (SV is the leading cardiometabolic physician and professor in Samoa). Additionally, feedback received from prior studies, and the Samoan MOH, indicated a need to determine how to resolve gaps within the NCD care cascade in this setting.

When engaging with the local community, how did you ensure that the informed consent documents and other materials could be understood by local stakeholders?

All of our consent documents, study instruments and advertising materials are provided in Samoan and English (Samoan is the official language, but English is spoken by many as it is taught in the education system). Additionally, because our local team has a long history of performing similar studies, they are skilled at providing and adapting their explanations of the study for their audiences.

Will the findings of the research be made available in an understandable format to stakeholders in the community where the study was conducted (e.g. via a presentation, summary report, copies of publications, etc.)? Please provide details of how this will be achieved.

Yes. Results of the study will be presented to participants and the Samoan Ministry of Health upon completion of the study. As used in the past, we plan to present the findings via a Samoan-language newsletter, that will provide the overall study findings. Additionally, as required by our local IRB, we will be constructing a final report of all findings to the Ministry of Health. Finally, we aim to be able to present our findings at the local university (National University of Samoa), where SV is a professor.

**Non-human subjects research using specimens/ animals collected as part of the study, or those housed in archival collections. Examples include archaeology, paleontology, botany and zoology.**

Did the permission you obtained from a local authority to perform the study include an agreement on access to outputs and benefit sharing? This may include procedures to enable fair distribution of the benefits and resources arising from the research performed. Please include any details of Prior Informed Consent and Benefit Sharing Agreements obtained. These may be required by field-specific regulations, for example the Convention on Biological Diversity (CBD) and the associated Nagoya Protocol.

N/A

If the material used in your study was imported, please A) provide the year it was imported and B) indicate whether permits were obtained to import/export the materials used, C) provide details of any permits obtained. If this information is not available, please indicate this.

N/A

If you used archival specimens, please state how the material used in your study was acquired by the institute it is held in and provide details of any permits obtained for the original excavations/ sample collection. If this information is not available, please indicate this.

N/A

How was the potential cultural significance of the materials collected in your study to local communities considered in your research design? Were Indigenous peoples and/or local researchers and institutions involved with archaeological excavations / collection of specimens? If so, please provide a description of their involvement.

N/A

If your manuscript includes photographs of human remains please indicate whether authors obtained permission from descendants or affiliated cultural communities to do so.

N/A
